# Single 5 μm diameter needle electrode block modules for unit recordings *in vivo*

**DOI:** 10.1038/srep35806

**Published:** 2016-10-25

**Authors:** H. Sawahata, S. Yamagiwa, A. Moriya, T. Dong, H. Oi, Y. Ando, R. Numano, M. Ishida, K. Koida, T. Kawano

**Affiliations:** 1 Department of Electrical and Electric Information Engineering, Toyohashi University of Technology, 1-1 Hibarigaoka Tempaku-cho, Toyohashi, 441-8580 Japan; 2 Electronics-Inspired Interdisciplinary Research Institute (EIIRIS), Toyohashi University of Technology, 1-1 Hibarigaoka Tempaku-cho, Toyohashi, 441-8580 Japan; 3 Department of Environmental and Life Sciences, Toyohashi University of Technology, 1-1 Hibarigaoka Tempaku-cho, Toyohashi, 441-8580 Japan; 4 Department of Computer Science and Engineering, Toyohashi University of Technology, 1-1 Hibarigaoka Tempaku-cho, Toyohashi 441-8580, Japan

## Abstract

Investigations into mechanisms in various cortical areas can be greatly improved and supported by stable recording of single neuronal activity. In this study, fine silicon wire electrodes (diameter 3 μm, length 160 μm) are fabricated by vapor–liquid–solid (VLS) growth with the aim of stabilizing recording and reducing the invasiveness on the measurement procedure. The electrode is fabricated on a modular 1 ×  1 mm^2^ conductive silicon block that can be assembled into a number of different device packages, for example on rigid or flexible printed circuit boards (PCB). After plating with a 5 μm diameter platinum black, the needle exhibits an electrical impedance of ~100 kΩ at 1 kHz in saline. The *in vivo* recording capability of the device is demonstrated using mice, and spike signals with peak-to-peak amplitudes of 200−300 μV in the range 0.5−3 kHz are stably detected, including single-unit activities in cortical layer 2/3. In addition, the device packaged with a flexible PCB shows stable unit recordings for 98.5 min (n = 4). Consequently, our modular, low-invasive needle electrode block devices present an effective route for single-unit recordings *in vivo*, as well as demonstrating adaptability in device design for a diverse range of experiments.

Stable recording of single-neuron activity, a phenomenon composed of sub-millisecond events, is vital to research aimed at understanding neuronal functions in brain tissue. Due to the source sizes of cell bodies, detection of signals with microscale spatial resolution is required, and thus needle electrodes with micrometer-scale diameters are commonly used. However, conventional electrodes tend to damage brain tissue as both the width (*e.g.*, >80 μm for silicon (Si) needles^1^,^2^,^3^,^4^ and >30 μm for metal electrodes) and diameter (*e.g.*, >40 μm for Si needles^5^,^6^,^7^) of these are relatively large, which makes stable, long-period recording of single-neuron activity difficult.

The use of fine needle electrodes would extremely beneficial to these studies, and to realize this we have designed and demonstrated needle-electrode arrays with tip diameters of <7 μm fabricated from Si microwires grown using the vapor–liquid–solid (VLS) process^8^,^9^; additionally, their *in vivo* recording capability has been demonstrated^10^. Immunohistochemical analysis indicates that these VLS growth-based microneedles are less invasive than the larger conventional electrodes (>40 μm diameter)^10^, suggesting that fine needle electrodes may be powerful experimental tools that reduce the invasiveness of procedures, and should improve the stability of long-period single-neuron recordings.

However, despite the clear advantages of using fine needle electrodes, previous devices made by this research group have been limited by their size: as the devices have been designed such that the interconnections and bonding pads are surface-sided (*i.e.*, on the same side as the needle electrode), they have required fabrication on Si substrates with dimensions greater than 5 × 5 mm^2 ^10. *In vivo* recordings with large substrate devices require a wide-area craniotomy (temporary removal of bone). In addition to the invasiveness of this procedure, a large craniotomy destabilizes brain vibration and pressure^11^, increases the risk of infection^12^, and damages the brain tissue and blood vessels underneath the bone^13^. Therefore, miniaturization of the device to allow the needle electrodes to approach various cortical areas *in vivo* is a priority, particularly when it comes to testing on animals with a relatively small brain tissue mass, such as rodents. In addition to device miniaturization, redesign of the wire-bonding section (>100 μm height) (see Supplementary Fig. S1a) is required as this hampers measurement by making contact with the brain tissue, preventing needle penetration, and also causes tissue damage from ischemia and friction (see Supplementary Fig. S1b).

In this study, we report an important step towards stable recording of single-neuron activity *in vivo*. This was achieved by solving the issues associated with (1) the large device substrate, and (2) the surface-sided bonding pad (1 mm height): to do this, we developed a manufacturing technique for a single-needle electrode on a small, heavily doped Si block module. Here, the conductive block module (1 × 1 mm^2^) acts as the vertical interconnection and, crucially, allows electrical connection from its backside. Although this concept is very simple, the device redesign solves significant issues with operation. In addition, this device concept enables it to be packaged in a number of different ways, thereby making it easy to adapt the design for numerous *in vivo* recording applications. The individual electrode modules can be assembled using different components, including conventional connectors and rigid or flexible printed circuit boards (PCBs), offering neuronal recordings from different sites based on application-dependent experimental conditions.

## Results

### Fabrication of the microneedle block module

**Figure 1:**
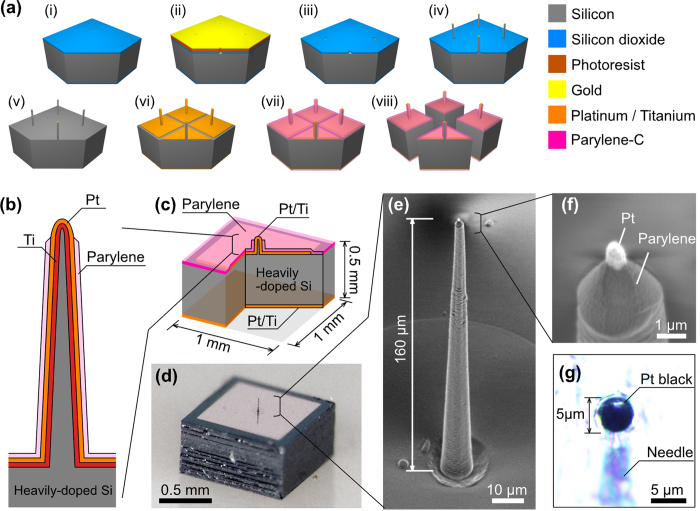
Fabrication process and structure of a single microneedle electrode block module. (**a**) Process steps: (i) Si oxidation, (ii) SiO_2_ etching and catalytic Au deposition, (iii) Au patterning by lift-off, (iv) VLS growth, (v) SiO_2_ and Au removal, (vi) Pt patterning by lift-off, (vii) parylene deposition and tip exposure, and (viii) separation of the block module by laser dicing. (**b**) Schematic of the cross-sectional structure of a silicon-needle electrode. (**c**) Schematic and (**d**) photograph of a Si-electrode block module. (**e**) SEM image of a needle-electrode shaft. (**f**) SEM image of the tip section. (**g**) Water-immersion optical microscope image showing the 5 μm diameter Pt-black needle tip formed by electroplating.

Figure 1a shows the fabrication process for the microneedle-electrode block module. Initially, a 400 nm thick silicon dioxide (SiO_2_) layer was thermally grown at 1000 °C. Then, the SiO_2_ layer at the needle site (10 μm diameter) was etched, exposing the Si surface for subsequent VLS growth. After placing a catalytic gold (Au) dot (200 nm thick and 6 μm diameter) on the exposed Si surface, the Si microneedle was fabricated by Au-catalyzed VLS growth of Si (see Methods)^14^.

After VLS growth, the Si needle and the substrate were metalized with platinum (Pt) by sputtering, such that the Pt layer and titanium (Ti) binder layer were 150 nm thick and 50 nm thick, respectively. These metal layers were patterned with gridlines for subsequent laser dicing of the Si substrate. Similarly, the backside of the Si substrate was metalized by the same method. After metal patterning, a highly biocompatible, insulating layer of parylene-C (1 μm thick) was deposited on the substrate, and the tip section of the needle was exposed by oxygen plasma. Finally, laser dicing was used to separate each section of the microneedle block module. Figure 1b–d show cross-sectional diagrams, schematics, and a photograph of a single-microneedle block module. Figure 1e,f show a microneedle with <1 μm diameter and 160 μm length fabricated at the center of a 1 × 1 mm^2^ Si block (0.5 mm thick and 1.2 mg), which acts as the vertical interconnection between the metalized microneedle and the backside Pt/Ti surface. To reduce the impedance of the needle electrode, the Pt needle tip was electroplated with a 5 μm diameter layer of Pt black^10^,^15^ after device packaging (see Methods) (Fig. 1g).

### Device packages

**Figure 2:**
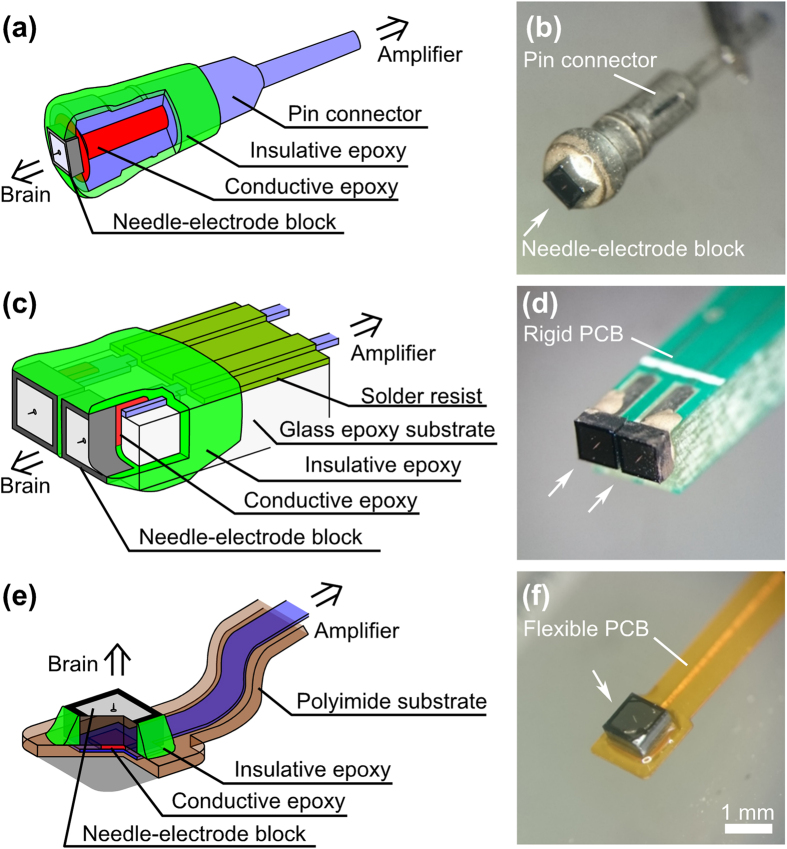
Variety of package designs for the needle electrode devices. (**a**) Schematic and (**b**) photograph of a single-pin connector needle module. (**c**) Schematic and (**d**) photograph of two needle block modules packaged with a rigid PCB. (**e**) Schematic and (**f**) photograph of a flexible FPC-packaged needle block module.

To demonstrate the versatility of the fabricated needle electrode block module, we used three different types of device package: ‘pin connector’ for the device electrical characterizations (Fig. 2a,b), ‘rigid PCB’ for multi-site *in vivo* recordings (Fig. 2c,d), and ‘flexible PCB’ to damp the animal’s physiological motion (such as pulsation) during the recording *in vivo* (Fig. 2e,f) (see Methods).

### Electrical characteristics of the microneedle module

**Figure 3:**
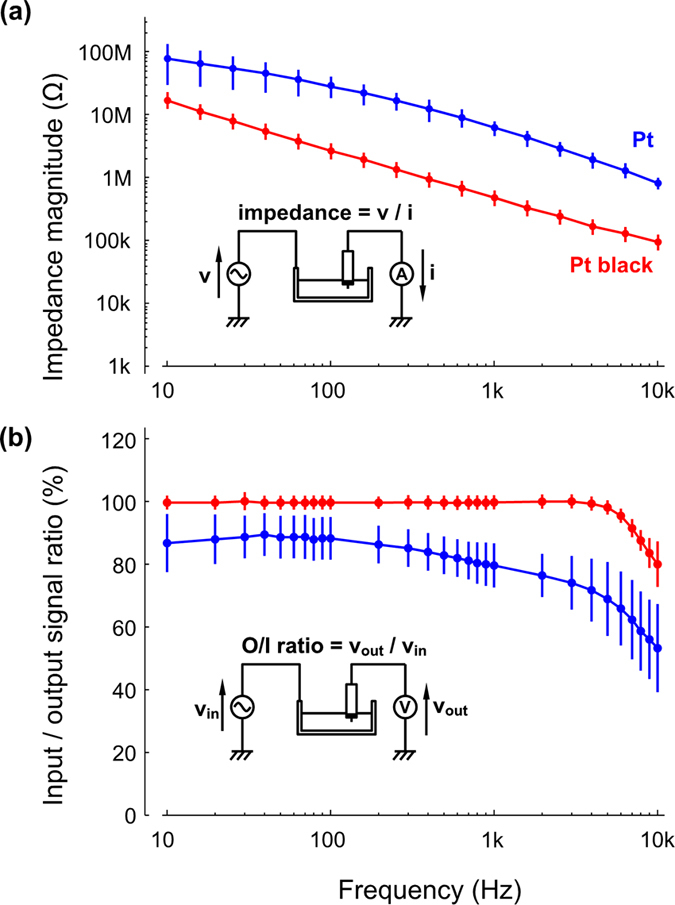
Electrical characteristics of needle-electrode modules measured in saline. (**a**) Impedance plot of pin-connector–packaged needle-electrode devices in room temperature saline (0.9% NaCl solution) at frequencies from 10 Hz to 10,000 Hz for the unmodified Pt-tipped electrode (blue) and the Pt black–tipped electrode (red). (**b**) Output/input signal amplitude ratios (O/I ratio) of the original Pt-tipped (blue) and the Pt black–tipped (red) electrodes taken from the test signal recordings. Test signals of 0.8 μV_p–p_ sinusoidal waves from 10 Hz to 10,000 Hz are applied to the saline. Averages and standard deviations of both impedance and O/I ratio characteristics are taken from six samples.

Figure 3a shows the magnitude of impedance of the pin connector microneedles (Fig. 2a,b). Before plating with Pt black, the Pt-tipped microneedle (Pt tip diameter <1 μm) exhibited an impedance of 6.5 ± 2.1 MΩ at 1 kHz (an average of six microneedle samples, denoted by the blue “Pt” plot in Fig. 3a), which is approximately equal to the frequency of a neuronal action potential. The output/input (O/I) ratios of the unplated Pt microneedle, measured with 80 μV_p–p_ test signals, exhibited a 20% signal attenuation at 1 kHz (blue “Pt” plot in Fig. 3b) due to the high impedance characteristics of the Pt tip, ~6.5 MΩ at 1 kHz, and the parasitic impedances of the recording system^10^,^16^.

The electroplating modification of the microneedle tip with a 5 μm diameter layer of Pt black (see the Methods section for details of the procedure) led to significant reductions in impedance^10^. The impedance for a Pt black–tipped microneedle was measured to be 450 ± 122 kΩ at 1 kHz (red “Pt black” plot in Fig. 3a), an order of magnitude lower than the unmodified Pt tip. The impedance reduction is predominantly attributed to the nanoporous structure of Pt black that enhances the surface area of the needle tip^15^. The impedance reduction simultaneously increases the O/I ratio (99.4% at 1 kHz), and thus the modified microneedles are able to carry out neuronal recordings without significant attenuation of the signal amplitude (red “Pt black” plot in Fig. 3b).

### *In vivo* neuronal recording from a mouse visual cortex

**Figure 4:**
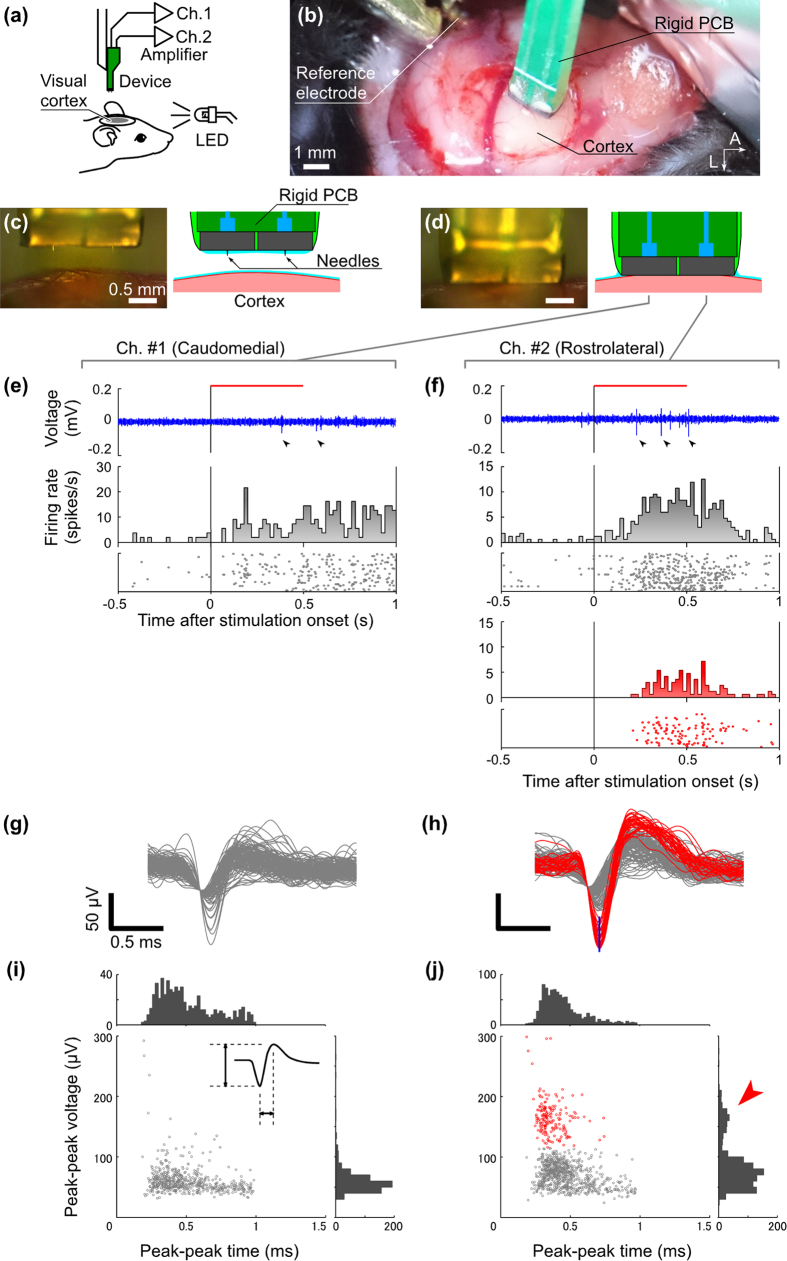
*In vivo* neuronal recording from the visual cortex of a mouse using a rigid PCB–packaged needle-electrode device. (**a**) Schematic showing the experimental setup. The head of an anesthetized mouse is fixed on the brain stereotaxic apparatus. The rigid PCB–packaged electrode device, which is held in place by a micromanipulator, is electrically connected to a head amplifier, and an array of white LEDs is located in the left visual field of the mouse for visual stimulation. (**b**) Photograph showing the placement of the electrode device over the cortical surface (A: anterior, L: lateral). Two needle electrodes are stereotaxically located on the primary visual cortex (V1) in the right hemisphere. Photographs and schematics of (**c**) before and (**d**) after the insertion of the needles into the cortex. Recorded signals from electrode (**e**) Chs. #1 and (**f**) #2. Waveforms in top panels show recorded signals taken from a single trial. Each horizontal red bar indicates the period of the visual stimulation (0−0.5 s). Middle (gray colored) and bottom (red colored) PSTHs and raster plots are residual and isolated signals by processing with a window discriminator, respectively. Enlarged waveforms of spike signals derived from (**g**) Chs. #1 and (**h**) #2. Superimposed 892 spikes from Ch. #1 and 532 spikes from Ch. #2 are respectively sorted with the time point where signals are detected using an amplitude threshold of −30 μV. Red colored waveforms are isolated into 158 spikes by the window discriminator (blue line segment in (**h**)), and gray waveforms represents other spikes. (**i,j**) Scatter diagrams and histograms of the peak-to-peak amplitude and peak-to-peak time (schematically illustrated in (**j**)) of these spikes.

To evaluate the performance of each packaged microneedle, neuronal signal recordings were performed using mouse cerebra *in vivo*. First, two microneedle modules packaged with a rigid PCB (Fig. 2c,d) were stereotaxically placed on the primary visual cortex (V1), then inserted into the rostrolateral and caudomedial regions in V1 via a 3 × 3 mm^2^ fenestra of cranium and dura mater (Fig. 4a,b) (see Methods). Here, the 160 μm long microneedles entirely penetrate the cortical tissue (Fig. 4c,d), and therefore the recording site of the needles corresponds to a depth of approximately 160 μm from the surface of the cortex: from known thicknesses of the cortical layers in mice, it is posited that the recording sites are located in either cortical layer 2/3, and the region will be referred to as layer 2/3 in the rest of the paper. Figure 4e,f show the signals delivered from caudomedial (Ch. 1) and rostrolateral (Ch. 2) needle-electrode channels. To apply visual stimulation, a light-emitting diode (LED) array consisting of nine white LED elements on a 20 mm wide substrate and a 10° visual angle was applied to the mouse’s left eye (contralateral eye from the recording hemisphere) for 500 ms. During visual stimulation, a single-trial sample simultaneously recorded by Ch. 1 and Ch. 2 (top panels for Ch. 1 and Ch. 2 in Fig. 4e,f) was observed to consist of spike-shaped waveforms (marks in Fig. 4e,f and enlarged waveforms in Fig. 4g,h) in the frequency band 0.5–3 kHz. By using an amplitude threshold of −30 μV, 892 spikes were detected from the rostrolateral channel (Ch. 1) and 532 spikes were detected from the caudomedial channel (Ch. 2) during the recording period (510 s). Raster plot diagrams and peristimulus time histograms (PSTHs) of these spike activities derived from both Ch. 1 and Ch. 2 (shown in the middle and bottom panels in Fig. 4f, 85 trials) show that the spike fires an excitatory response to the visual stimulation [*p* = 0.28 × 10^−6^ for all detected waveforms from Ch. 1, *p* = 0.34 × 10^–5^ for Ch. 2 by Wilcoxon signed-rank test between baseline (−500 ms to 0 ms) and response (0 ms to 500 ms) periods]. Figure 4g,h show an enlarged view of the waveforms of the spikes detected from each channel. The waveforms consist of negative and positive peaks, similar to waveforms of extracellularly measured action potentials. In the superimposed spike waveforms from the rostrolateral channel, two groups of peaks are present, one with large amplitudes and the other with small amplitudes. From these, 158 large spikes (red traces in Fig. 4h) were isolated using a window discriminator (80–130 μV at 0.15 ms after triggering, blue line segment in Fig. 4h). For a detailed analysis, the amplitude and period from peak-to-peak of all spike waveforms in each channel are plotted two-dimensionally in scatter diagrams and histograms, displayed in Fig. 4i,j.

Figure 4j includes a cluster of red plot points, which represent the red-colored waveforms with larger amplitudes in Fig. 4h. The cluster is well isolated from the other cluster (gray plot) with a boundary of approximately 120 μV_p–p_. In addition, the histogram of the peak-to-peak amplitude of red and gray plots indicates that a bimodal distribution is present. Consequently, it can be surmised that the cluster of red plots indicates single-unit activity. The isolated unit activity implies a significant excitatory response (*p* = 0.15 × 10^−11^ by the Wilcoxon signed-rank test, 0–500 ms from the stimulation onset) and a minimum latency of 201 ms. The median latency across all trials was calculated to be 340 ms. These latencies are longer than the electrical and optoelectrical noise due to the onset of LED driving (<1 ms, data not shown). Therefore, it can be concluded that this isolated unit originates from physiological phenomena.

### *In vivo* neuronal recording from a mouse somatosensory cortex

**Figure 5:**
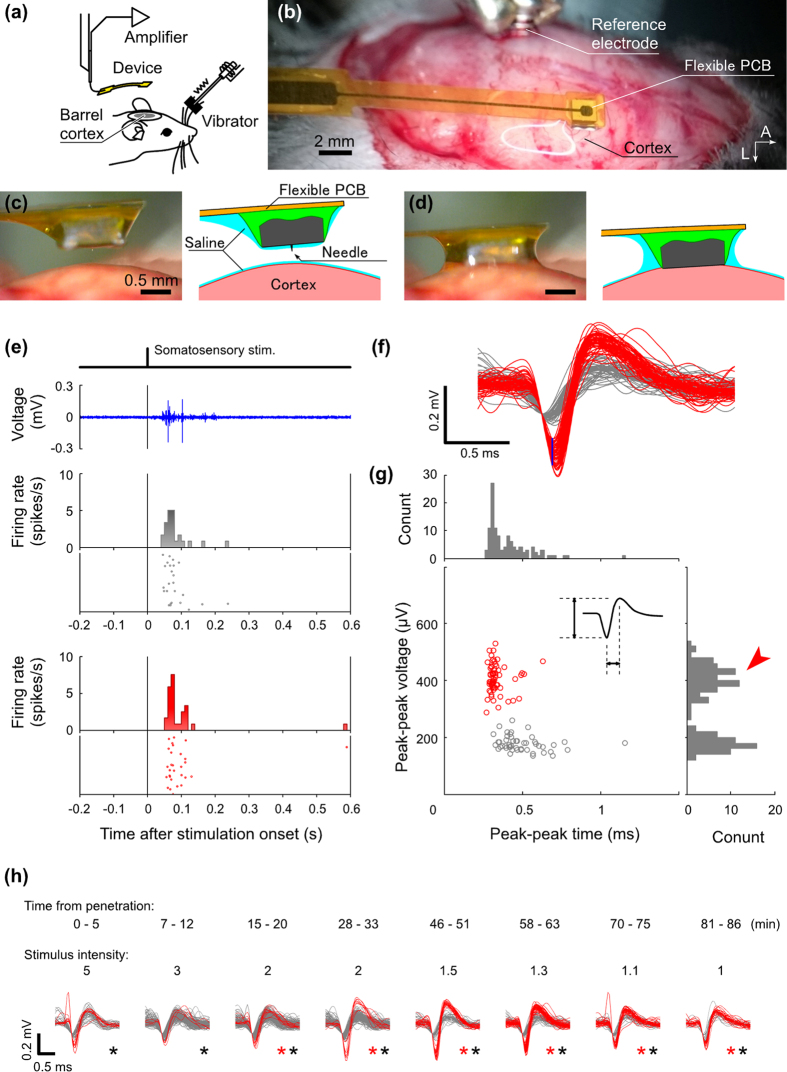
*In vivo* recordings from the somatosensory cortex of a mouse with a flexible PCB–packaged electrode device. (**a**) Schema of the experimental setup. The head of the anesthetized mouse is immobilized on the brain stereotaxic apparatus. The flexible PCB–packaged electrode device, which is held in place with a micromanipulator, is connected to a head amplifier. The mouse’s principal whiskers are mechanically stimulated with an electromagnetic vibrator via a metal wire. To drive the stimulator, an electrical pulse with a 2 ms duration is applied at 6 s intervals for a 360 s recording period (60 trials). (**b**) Photograph showing the electrode placement over the cortical surface. A needle electrode is stereotaxically located on the barrel field (S1B) in the right hemisphere. Photographs and schematics of (**c**) before and (**d**) after the needle insertion into the cortex. (**e**) Recorded signals with the needle electrode. The waveform in the top panel shows recorded signal of a single trial. The spike in the black line in the range 0–2 ms indicates the pulse signal to drive the vibrator for the somatosensory stimulation. Middle (gray colored) and bottom (red colored) PSTHs and raster plots represent the residual and isolated signals by a window discriminator. (**f**) Enlarged waveforms of the spike signals. Superimposed 54 spikes are sorted with the detected time point with an amplitude threshold of −100 μV. Red waveforms are isolated into 29 spikes by the window discriminator (blue line segment in (**f**)), and gray waveforms are other spikes. (**g**) Scatter diagram and histograms of the peak-to-peak amplitude and peak-to-peak time of these spikes. (**h**) Waveforms of these spikes in each 5 min recording segment (total experiment time of 86 min, 8 segments in total) after the needle penetration. Red and black asterisks indicate significant increases in the firing-rate responses to somatosensory stimuli (*p* < 0.01, n = 120 trials, Wilcoxon signed-rank test). Spikes shown in (**f**) are the same as data of sixth period (58–63 min).

Secondly, a flexible PCB–packaged microneedle (Fig. 2e,f) was stereotaxically inserted into the barrel field in the primary somatosensory cortex (S1B) (Fig. 5a,b) (see Methods). During needle penetration, the needle device was slowly positioned over the brain with the manipulator system (~50 μm/s). Once the surface of the needle device made contact with the brain surface, the needle fully penetrated the tissue within 0.1 s (Fig. 5c,d, see Supplementary Movie S1), and signal recording commenced. The penetration force is likely due to the surface tension of the saline solution between the hydrophilic parylene film coating the device module and the cortical surfaces; however, further quantitative discussion is necessary to clarify the precise origins of the penetration force.

Spike-shaped waveform signals were recorded after needle penetration. Figure 5e shows spike-shaped waveform signals acquired during a single trial in which the mouse’s principle whiskers underwent mechanical stimulation. During the 86 min recording period, well-isolated spike signals were obtained between 28 min and 63 min. Here, the signals generated in the period between 46 min and 51 min are discussed further. Initially, spikes were identified from the original waveforms using a threshold of 100 μV, followed by isolation of single spikes (red traces for single spikes and gray traces for others in Fig. 5f) by using a discriminating window [180–280 μV at 0.15 ms (blue line segment in Fig. 5f)]. The middle and bottom panels in Fig. 5e show the PSTHs and raster plot diagrams for the large (red) and small (gray) amplitude spikes, respectively. The spike activity is in response to somatosensory stimulation (*p* = 4.2 × 10^−7^) with a latency of 45 ms. Figure 5g is a scatter diagram of the peak-to-peak amplitude and the period of these spikes. As was the case for Fig. 4j, the histogram indicates a bimodal distribution of the spike amplitude.

Figure 5h shows all the spikes registered during the 86 min recording period (eight segments, each with a 5 min duration). During the recordings, several units were detected with the arbitral criterion. The signals analyzed in Fig. 5e–g are taken from the sixth recording segment (58–63 min in Fig. 5h). Red and black asterisks indicate that there were significant increases in the firing-rate responses to somatosensory stimuli (*p* < 0.01, n = 120 trials, Wilcoxon signed-rank test). Some spikes were immediately recorded after the needle penetration, as shown in the first recording segment (0–5 min in Fig. 5h). The longest period of the holding unit was up to 86 min (81–86 min in Fig. 5h). In addition, using additional three different electrode devices (Supplementary Fig. S2a), the averaged holding times of single unit signals was 98.5 ± 16.9 min (n = 4). In comparison, conventional metal needle electrodes penetrated at the same depth of 150–200 μm (see Methods) exhibited the holding time of 8.33 ± 1.44 min (n = 3) (Supplementary Fig. S2b), which was significantly shorter than our electrode devices (*p* < 0.01, Student’s *t*-test).

## Discussion

In this study, as a step towards stable recording of single-neuron activity *in vivo*, we solved issues relating to (1) the large device substrate size and (2) a surface-sided bonding pad with a disruptive height (~1 mm), by proposing the single-electrode block module device on a conductive 1 ×  1 mm^2^ Si substrate. This configuration is smaller than previous devices demonstrated by this group and possesses a backside connection, thereby eliminating the need for a surface-sided bonding pad. Needle electrodes with a diameter of 5 μm are successfully fabricated on the blocks, and the modular nature of the device allows it to be adapted for different *in vivo* recordings, as demonstrated by the assembly of three different device packages. Using the packaged devices, well-isolated single-unit activities are recorded from superficial layer 2/3 of mouse’s visual and somatosensory cortices.

### Device miniaturization and backside connection

We have demonstrated that the use of a fine needle electrode with a tip diameter of <7 μm reduces tissue damage^10^,^17^. In addition to investigating the effects of using a fine needle, this work also examined the geometry of the device substrate. The backside electrical connection of the block module minimizes the device surface area to 1 × 1 mm^2^, which is 24 times smaller than our previously proposed devices (>8 × 3 mm^2^)^10^. In addition, the backside electrical connection eliminates the need for surface-sided bonding wires (~1 mm height) that were present on our previous device^10^, thus making the needle-side device surface much smoother and further reducing tissue damage. Advantages of this block substrate design include a reduction in the craniotomy and durotomy areas required (due to the small surface area of the device) and the adaptability of the device for a number of different *in vivo* recording applications.

For the backside electrical connection, we used a doped Si block with low resistivity. The Si block (<0.2 Ω·cm, 1 × 1 mm^2^ surface area, and 500 μm height), which acts as the vertical interconnection, has a resistance of <0.1 Ω, which is ~ 4.5 × 10^4^ times lower than the needle electrode impedance (450 kΩ at 1 kHz, Fig. 3a). The parasitic impedance of the parylene-C layer over the block surface can be determined using the film thickness: in order to attain high O/I signal amplitude ratios of the recording system, a 1 μm thick parylene-C layer was formed over the block surface, resulting in an impedance of 5.7 × 10^6^ Ω at 1 kHz. The impedance is 12.7 times larger than the needle-electrode impedance (450 kΩ at 1 kHz), while O/I ratios of >90% in the range 10 Hz–4 kHz were observed in the test signal recordings (Fig. 3b).

In contrast with conventional electrode devices, the depth of the recording site within the tissue using this needle electrode device is very consistent. Conventional needles possess a larger diameter (for example, previous studies report using Si needles with 40 μm width^5^,^6^,^7^ and diameters of 80 μm^1^,^2^,^3^,^4^), and consequently deform the tissue during penetration due to the relatively large force required to puncture the surface; however, the fine needle electrodes possess a diameter of 5 μm diameter, and therefore encounter less physical resistance from the tissue on insertion. Consequently, the penetration process occurs smoothly and the needle reaches its target penetration depth very rapidly (<0.1 s, Fig. 5c,d, see Supplementary Movie S1). Moreover, as the needle is inserted into the tissue, the device stops when the Si substrate makes contact with the cortical surface, *i.e.* at full needle penetration. As a result, this prevents over-penetration of the tissue by the needle, and the penetration depth of the needle is therefore determined by its length (160 μm).

### Reduction of invasiveness

In terms of physical effects, the small size of the device block carries a number of advantages, including less destabilization of intracranial pressure^11^, decrease in the risk of infection^12^, and a reduction in damage to the brain tissue and blood vessels underneath the bone^13^ as the required craniotomy and durotomy areas are smaller than for other devices. Destabilization of intracranial pressure has been shown to negatively affect brain activity in short-term acute experiments, and in long-term experiments, a pathological condition has been shown to arise from the surgery. In this procedure, we extracted a ~3 × 3 mm^2^ section of dura mater (2−5 mm posterior and 1−4 mm lateral to bregma) for the 1 × 1 mm^2^ Si block; the craniotomy area is reduced to such an extent that a ~5 μm diameter slit can be prepared in the dura mater using microscissors prior to needle penetration. Although the durotomy area in this study was ~ 3 × 3 mm^2^, this is much reduced from our previous work, in which the dimension of the device was > 5 × 5 mm^2 ^10. In addition, the device surface, which makes direct contact with the brain tissue, is smooth, flat, and covered with a chemically stable and biocompatible parylene-C polymer layer: as a result, the mechanical stress on the tissue (such as ischemia due to local pressure along with damage to the pia matter and blood vessels due to friction) and biochemical effects can be minimized. Along with the size reduction, the lightweight design of the block module (1.2 mg) and the elasticity of the polyimide PCB in the flexible package (Fig. 2e,f) also helps reduce the mechanical stress. The optimization in device design reduces the overall invasiveness of the measurement procedure, thereby maximizing the benefits of using fine needle electrodes over conventional electrodes.

### Design variability in the packaging

In electrophysiological experiments, electrodes must be designed and adapted to suit different experimental conditions (*e.g.*, acute or chronic experiments) and recording targets (*e.g.*, brain regions and cortical layers). Our proposed electrode block module device is highly adaptable, as exemplified the different device packages that may be assembled, as shown in Fig. 2. The single-pin connector design (Fig. 2a,b) is the simplest, and can be manipulated in a similar fashion to conventional single-needle electrodes (*e.g*., metal electrode and glass pipette).

A rigid PCB package composing of two or more electrode block modules would be suitable for multisite recordings in future experiments. Although our block module has a surface area of 1 × 1 mm^2^ that requires an inter-electrode spacing of ≥1 mm, the block module can further be miniaturized (*e.g*., to 0.2 × 0.2 mm^2^) by using laser dicing. The ability to use small block modules enables the assembly of high-density electrode block arrays, *e.g.*, ~250 μm spacing (compared to a Utah array that has spacings of 400 μm). For assembly of multiple Si block mini-modules, it is possible to use a rigid PCB that allows for multiple interconnections; moreover, the size of the rigid PCB does not increase when a narrow interconnection (*e.g*., <100 μm in width) is used.

By adding the device blocks on a flexible PCB, it is possible to damp tissue vibration during recording that arises from the animals’ breathing and pulse, providing a simple route to enable acute and chronic long-period measurement. In addition to the advantages conferred by the different PCB packages, the arrangement of the electrode arrays may be modified by changing the design of PCB (*e.g*., increasing the number of electrodes and altering the arbitrary spacing between electrodes). As with the rigid PCB, multiple high-density electrode blocks can also be assembled on the flexible PCB by using smaller electrode blocks and multiple narrower interconnections.

### Single-unit recording from the superficial layer of a mouse cortex

In previous studies, conventional electrode devices have been used in single-unit recording from large neurons, such as the pyramidal cells in cortical layer 5. As these electrodes are relatively large, the sites for single-unit recordings from small neurons such as the neurons in layer 2/3 are also somewhat larger than ideal (~20 μm in diameter)^18^. Although conventional metal electrodes are often used to isolate cells in layer 2/3, the smaller microelectrodes used in this study are potentially better suited to isolating activity of single cells in the cortex. Neurons within layer 2/3 are currently being studied because they correspond to highly ordered information processing in high-density neuronal circuits^19^,^20^,^21^.

Generally, single-unit recording from regions with high neuron density is difficult because the electrode site needs to be close to the target neuron but also sufficiently far away from other neurons. Additionally, the electrode tip should be small in comparison to inter-neuron distance. These conditions are crucial to produce a significant difference in the signal amplitude between the target and the other neurons, as well as isolating the single unit.

The needle electrodes used in this study have a length of 160 μm, which is sufficient to reach a mouse’s layer 2/3. Since a mouse’s layer 1 consists of only a few neurons, it can be deduced from the unit-activity recordings that the electrode reaches layer 2/3. In addition, the thicknesses of layer 1 and layer 2/3 are about 100 μm and 200–300 μm, respectively^18^,^22^, therefore eliminating the possibility of signal detection from layer 4. In order to evaluate the actual depth of needle penetration, possible methods include making lesion marks in the tissue, although this procedure was not carried out in this study.

The rigid and flexible PCB microneedle packages were used to record signals from the V1 and S1B of the mice, respectively, and the spike signals acquired included single-unit activities. It is known that a single unit reflects the activity of a single neuron^23^,^24^, and the timings of the recorded spikes were within the range of variation of response latencies of mouse’s V1^25^ and S1B^26^ that depend by the stimulus conditions. Given the length of the needle electrodes, it can be confirmed that the isolated unit signals are recorded from the layer 2/3. Although the mouse layer 2/3 contains small neurons (~10 μm diameter) with a higher density (inter-neuron distance of ~20 μm) in comparison with other brain areas and other mammals^18^, the 5 μm diameter electrodes used in this study enable single-unit recordings, as demonstrated by the *in vivo* recordings detailed in Figs 4 and 5.

### Long-period single-unit recording

The flexible PCB provides cable elasticity between the manipulator and the needle module during penetration and recording. Using the flexible PCB–packaged device, single-unit activities were recorded for 98.5 min (n = 4). Conventional single-unit recordings require precise positioning of the electrode and a waiting time to achieve a stable holding unit. In order to record for a long holding time, additional procedures are required to eliminate the effect of pulsation, such as tracking neurons and inserting an agar layer between cortical surface and the electrode^27^. On the other hand, the needles used in this study offer immediate, stable holding of single units after penetration, and single-unit activity can be observed long periods (>86 min) without these additional procedures.

The longest single-unit activity recording time in this study (98.5 min) is substantially greater than our previous works on single-unit recording (1.7 min for a rat)^10^. In the previous studies, stable *in vivo* unit recording was complicated by brain vibrations: this was attributed to the previous experimental setup in which the electrode was rigidly fixed to the manipulator^10^, thus causing fluctuations in the relative distance between the target neurons and the electrode tip. In this study, the addition of the flexible PCB unit to mitigate the effects of brain vibration contributed to the success of single-unit recording over long periods. Overall, the stability of recording is attributed to the size of the 1 × 1 mm^2^ device substrate, the 5 μm diameter needle, and the mechanical flexibility of the device package.

The flexible needle electrode device reported here should enhance unit recordings *in vivo*. As reported in studies into neuronal recordings using electrocorticography electrodes^28^,^29^, flexible planar electrodes can be placed on the surface of soft brain tissue: these adapt to the brain surface and can damp pulse vibration. In addition, it is possible to place the electrode on narrow subdural or intrasulcal gaps in large animals^30^. Such flexible planar electrodes enable the detection of local field potentials, which are associated with large neuronal population levels. In contrast, the flexible microelectrode devices reported here are applicable to stable, single-unit recording from cortical regions that cannot be accessed by conventional needle electrodes. Furthermore, multi-channel single-unit recording can be realized by assembling an array of needle blocks on a flexible PCB with multiple interconnections.

In summary, we demonstrate needle electrodes with a 5 μm diameter on 1 × 1 mm^2^ Si blocks for stable recording of single-neuron activity *in vivo*. The electrode block module design is also shown to offer a strong degree of flexibility, allowing it to be adapted for numerous *in vivo* experiments. Our needle-electrode block devices can simply be placed on a small area of brain tissue—in this study, on mouse cerebrum cortices—while high-quality signals of a single unit can be stably recorded for a long period (98.5 min). As confirmed during these experiments, our electrode device reduces the total invasiveness of the measurements with respect to brain tissue *in vivo*, demonstrating the suitability of needle-electrode devices in neurophysiology.

## Methods

### Fabrication of the microneedle

A microneedle electrode was fabricated on a Si block (Fig. 1). In order to use the Si block as a vertical interconnection, a 0.5 mm thick Si substrate with a low resistivity of <0.2 Ω·cm was employed (<0.1 Ω block resistance for 1 × 1 mm^2^ design). A catalytic Au dot (6 μm diameter, 200 nm thick) was placed on the Si surface by evaporation and photoresist-based lift-off processes. The Si microneedle was fabricated on the Si substrate by Au-catalyzed VLS growth in a gas-source molecular beam epitaxy (GS-MBE) chamber using disilane (Si_2_H_6_) as the Si gas source; growth was carried out at a gas pressure of 4.9 × 10^−3^ Pa and a temperature of 700 °C^14^ for a duration of 185 min, leading to the formation of a Si microwire ~160 μm in length. Due to the Si_2_H_6_-mediated Si growth, the surface of the SiO_2_/Si substrate was covered with a poly-silicon layer, which was subsequently removed by inductively coupled plasma reactive ion etching. Afterwards, the exposed SiO_2_ layer was removed by wet etching with a buffered hydrofluoric acid solution. For device metallization, the exposed Si needle and the substrate were coated with Pt/Ti layers [Fig. 1a(vi)].

### Needle tip modification with Pt black

To reduce the impedance of the needle electrode, the Pt tip of the needle was electroplated with Pt black after assembly of the device package. On lowering the Pt-tipped needle electrode into the plating solution [10 g H_2_PtCl_6_∙6H_2_O, 0.1 g Pb(CH_3_COO)_2_∙3H_2_O, and 300 mg diluted water], a negative voltage of −400 mV was applied to stimulate the growth process, using a Pt wire as the counter electrode^10^,^15^. A 5 μm diameter Pt black–plated tip was formed after electroplating for ~4 s, with the plating current reaching a value of 400 nA (Fig. 1f). The electroplated Pt black has a high enough binding force to the Pt-tipped needle, as confirmed in the cycle tests of needle penetration into and extraction from a tissue more than 20 times without detachment of the platinum black from the tip.

### Device packaging

For electrical characterization of the fabricated devices, the microneedle block modules were packaged with a single-pin connector (1.4 mm diameter and 7.3 mm long, as shown in Fig. 2a,b). Here, the backside electrode of the Si block (coated with Pt/Ti) was connected to the female side of the pin connector with conductive glue (CircuitWorks Conductive Epoxy, Chemtronic, USA). The surfaces of the Si block, conductive glue, and the pin connector were all covered with electrically insulating glue (Araldite-Rapid, Huntsman Advanced Materials, USA).

For multi-site *in vivo* recordings, microneedle block modules were packaged with a rigid, glass epoxy–based PCB, which was equipped with multisite leads (Fig. 2c,d). The use of such a rigid PCB allowed for precise positioning of the needle using a micromanipulator prior to penetration. The rigid PCB (1.4 mm thick, 2.1 mm wide, and 25 mm long) used copper (Cu) interconnections and an electrically insulating solder resist, and the two block modules were mechanically fixed onto the sidewall of the PCB and electrically connected to the Cu interconnections using conductive epoxy glue. Then the sidewalls of the block modules, conductive epoxy glue, Cu, and interconnections were covered with insulating epoxy glue. Finally, Au pins were soldered to the counter side of the PCB in order to make a connection with the recording amplifiers.

The other PCB package used in this study was a polyimide-based flexible PCB, which was able to damp physiological motion during *in vivo* recording (Fig. 2e,f). The PCB had an 18 μm thick Cu interconnection embedded in the polyimide film (12.5 μm thick, 2.0 mm wide, and 20 mm long). Part of the polyimide layer was opened to assemble the block module. The block module was mechanically secured to the surface of the flexible PCB and electrically connected to the Cu interconnection using conductive epoxy glue. The sidewalls of the block module and conductive epoxy were then covered with the insulating epoxy glue, while a Au pin connector was soldered to the other end of the PCB.

### Measurement of electrical characterizations

The impedance of the needle block module’s electrolyte/electrode interface was measured in a phosphate-buffered saline (PBS) bath at room temperature using an impedance analyzer (Model 1260A Impedance/Gain-Phase Analyzer, Solartron Analytical, UK). A test sinusoidal wave with an amplitude of 100 mV_p–p_ was applied to the PBS bath via a silver/silver chloride (Ag/AgCl) counter electrode^10^.

The output/input (O/I) signal amplitude ratios of the microprobe were measured by applying test signals to the block module in the PBS bath using a programmable signal control system [RZ2, TDT, Alachua, USA, input impedance of a head amplifier (ZC64) = 10^14^ Ω]. The test signals were 80 μV_p–p_ sinusoidal waves (10 Hz–10 kHz), which were applied to the PBS bath via the Ag/AgCl electrode^10^.

### *In vivo* recordings

To prepare the animals for the *in vivo* measurements, mice (male, 20–25 g) were anesthetized by intraperitoneal injection using urethane (50 μl of 30% solution per 10 g body weight). After the head of a mouse was fixed with stereotaxic apparatus (SR-50, Narishige, Tokyo, Japan), parts of the cranium and dura mater of the recording sites were removed. During the recording, the brain surface was kept wet through dropwise addition of saline. The needle modules were packaged with either the rigid or flexible PCB and attached to a micromanipulator (MO-10, Narishige) to control needle placement. The recording site was stereotaxically defined, after which the microneedle was inserted to the mouse’s brain; an optical microscope was used to confirm that the needle was fully inserted into the brain tissue. All experimental procedures were approved by the Committee for the Use of Animals at Toyohashi University of Technology, and all animal care followed the Standards Relation to the Care and Management of Experimental Animals (Notification No. 6, March 27, 1980 of the Prime Minister’s Office of Japan).

For visual response recordings, two microneedle modules packaged with a rigid PCB (Fig. 2c,d) were positioned over the primary visual cortex (V1) on the right hemisphere via a fenestra of cranium and dura matter (2−5 mm posterior and 1−4 mm lateral to bregma). The two needles were inserted into the rostrolateral (4 mm posterior and 2.5 mm lateral to bregma) and caudomedial (4.7 mm posterior and 1.3 mm lateral to bregma) sites in V1^22^. The visual stimulation was applied by a LED array (nine white LED elements on a 20 mm wide substrate and a 10° visual angle) to the mouse’s left eye for 500 ms. After an inter-trial interval of 1500 ms, the next stimulation was applied. Trials were repeated 160 times. The LED array was driven by a processing system (RZ2, Tucker-Davis Technologies, Alachua, USA). The timing pulse signals of these stimulations were synchronized to acquire neuronal signals.

For somatosensory response recordings, the microneedle/flexible PCB package was used (Fig. 2e,f), and the electrode was stereotaxically inserted into the barrel field in the primary somatosensory cortex (S1B) (1 mm posterior and 4 mm lateral to bregma^22^) on the right hemisphere via the fenestra of cranium and dura matter (3–4 mm posterior and 2–3 mm lateral to bregma). The connector end of the flexible PCB was attached to the manipulator during both needle insertion and device operation. Because the electrode end was not firmly secured, the needle block module and the manipulator were flexibly connected (Fig. 5a,c). After needle insertion, the principal whiskers of the mouse were mechanically stimulated to activate the S1B neurons by an electromagnetic vibrator, which was driven by 5 ms duration pulse signals from the processing system (RZ2, Tucker-Davis Technologies, Alachua, USA). The stimulus intensity was manually modified to observe cell activity with an inter-stimulus interval of 3 s over a long period of up to 120 min. The timing pulse signals of these stimulations were synchronized to acquire neuronal signals.

To compare the recording capability of our electrode device, we also used conventional metal needle electrodes [Tungsten Electrode (1 MΩ at 1 kHz), World Precision Instruments, USA]. The metal electrode was inserted at the same depth of our electrode of 150–200 μm using a micromanipulator (MO-10, Narishige). During the single unit recording, position of the manipulator was fixed without tracking unit signal.

In signal acquisition and processing, signals recorded from the microneedles were differentially amplified using a head amplifier (ZC64, Tucker-Davis Technologies, 1 × 10^14^ Ω input impedance) with filters (0.35 Hz for low-cutoff and 7.5 kHz for high-cutoff). As a signal reference electrode, a stainless steel screw was drilled into the skull over the cerebellum. Following signal amplification, signals were routed to a preamplifier/digitizer (PZ2, Tucker-Davis Technologies) and a digital signal processing module (RZ2, Tucker-Davis Technologies). Digital data were stored on a hard disk in a Windows PC with a sampling frequency of 25 kHz.

Spike activity was detected offline from the filtered signals (0.5−3 kHz, second-order Butterworth filter) by thresholding on MATLAB (Mathworks, USA). To analyze unit activities, the triggered signals were isolated with a window discriminator. For each unit activity, a peristimulus time histogram (PSTH) was calculated (bin width = 20 ms, spike counts within any bin were summed across trials). The significance of sensory responses was tested by the student’s *t*-test between the response (0 ms to 100 ms) and the baseline (−100 ms to 0 ms) periods.

## Additional Information

**How to cite this article**: Sawahata, H. *et al*. Single 5 µm diameter needle electrode block modules for unit recordings *in vivo. Sci. Rep.* **6**, 35806; doi: 10.1038/srep35806 (2016).

## Supplementary Material

Supplementary Information

Supplementary Information
