# Supplementary material for: Single 5 μm diameter needle electrode block modules for unit recordings in vivo
Source: Sci Rep. 2016 Oct 25;6:35806. doi: 10.1038/srep35806 (PMC5078846; doi:10.1038/srep35806)
Supplement: Supplementary Information [file srep35806-s2.pdf]

Single 5-μm-diameter needle-electrode block modules for unit recordings *in vivo*

Supplementary information

H. Sawahata, S. Yamagiwa, A. Moriya, T. Dong, H. Oi, Y. Ando, R. Numano, M. Ishida, K. Koida, T. Kawano

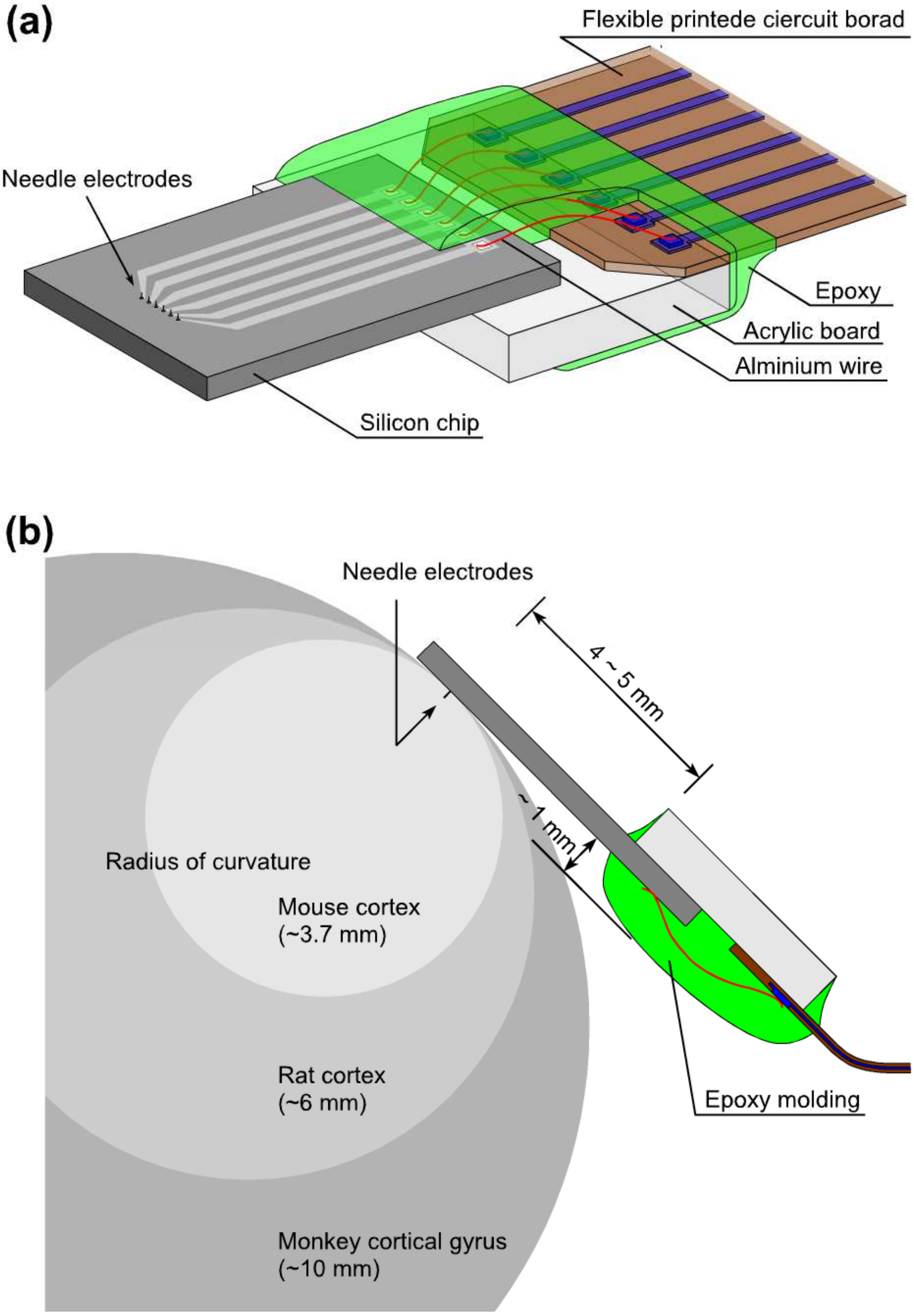

Supplementary figure S1 (a) Schematic of a needle-array substrate (> 8 mm × 3 mm) with bonding wires. These bonding wires are covered with electrically isolating glue

(total height of the bonding section is > 100 µm) for animal experiments. (b) Schematic illustration of device placement over a spherical sample, which represents an animal brain (coverture radii of 3.7 mm for mouse and ~6 mm for rat cortices, and ~10 mm for cortical gyrus of monkey).

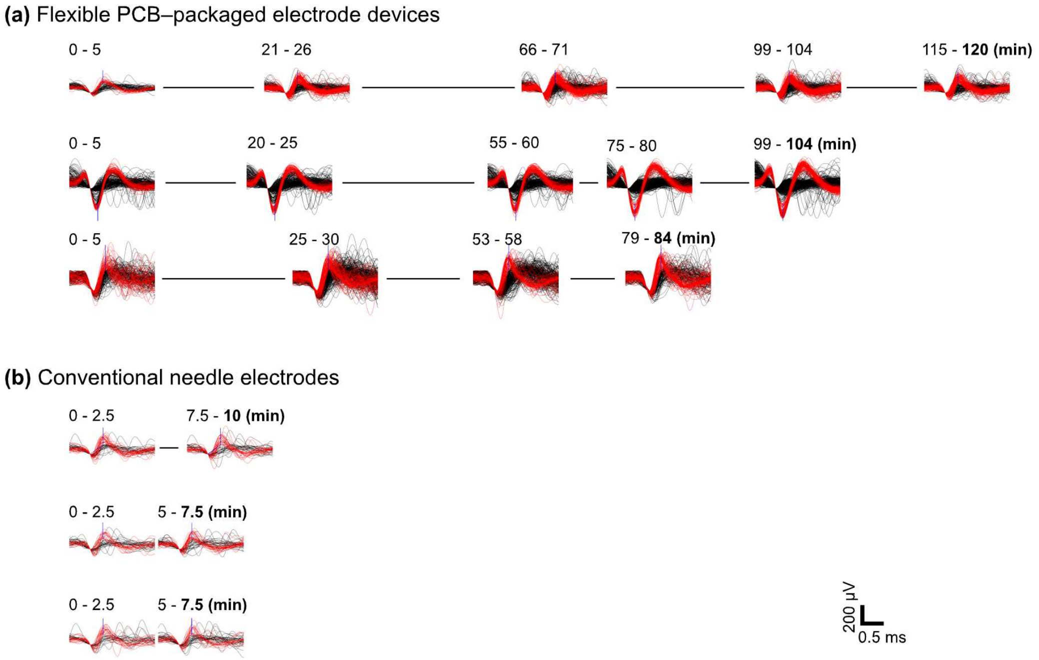

Supplementary Figure S2

(a) Waveforms of single unit signals recorded with three flexible PCB–packaged electrode devices. (b) Waveforms of single unit signals recorded three conventional needle electrodes. Red waveforms were detected with window discriminator (blue line segment).

Supplementary movie S1 Microscope observation of a needle penetration into a mouse cerebral cortex. The movie also includes the device height–time curve, indicating that the device is gradually moved downward to reach the brain surface (0–2.5 s), the device makes contact with the brain surface (> 2.5 s), and the needle immediately penetrates within < 0.1 s (2.6–2.7 s).
